# A novel HIV vaccine targets the 12 protease cleavage sites

**DOI:** 10.1186/1742-4690-9-S2-P304

**Published:** 2012-09-13

**Authors:** M Luo, D Tang, R Capina, X Yuan, C Prego, JC Pinto, M Alonso, C Barry, R Pilon, C Daniuk, J Tuff, S Pillet, D La, T Bielawny, C Czarnecki, P Lacap, H Peters, G Wong, M Kimani, C Wachihi, J Kimani, TB Ball, P Sandstrom, G Kobinger, FA Plummer

**Affiliations:** 1 National Microbiology Laboratory, Winnipeg, Canada; 2 University of Santiago de Compostela, Santiago de Compostela, Spain; 3 University of Nairobi, Nairobi, Kenya

## Background

The protease of HIV-1 is a small 99-amino acid aspartic enzyme mediating the cleavage of Gag, Gag-Pol and Nef precursor polyproteins. The process is highly specific, temporally regulated and essential for the production of infectious virions. A total of 12 proteolytic reactions are required to generate a viable virion. Therefore, a vaccine targeting the 12 protease cleavage sites(PCS) could be effective. The PCS of HIV-1 are highly conserved among major subtypes, direct immune responses against these sites would yield several advantages. First, the immune response could destroy the virus before its establishment in the host. Second, the vaccine could force the virus to accumulate mutations eliminating the normal function of the HIV protease. Third, restricting the immune responses to these sites can avoid distracting immune responses that often generate unwanted inflammatory responses, induce excess immune activation, and attract more targets for HIV-1 infection, establishment and spread.

## Methods

We have conducted a pilot study to investigate the feasibility and effectiveness of this approach. The recombinant VSV-peptides were used to immunize cynomolgus macaques and nanopackaged peptides were used to boost the immune response to the 12 PCS of SIVmac239. The controls and immunized macaques were repeatedly challenged intrarectally with an increased dosage of SIVmac239.

## Results

Results showed that antibody and T cell responses to the 12 PCS can protect macaques against higher dosage of SIVmac239 challenge (p=0.0005, R=0.8005) and the vaccine group maintains significantly higher CD4+ counts (p=0.0002) than the controls weeks after being infected. Population coverage analysis showed that this approach can be applied to >95% populations in the world.

## Conclusion

A vaccine targets the 12 protease cleavage sites is a viable approach for HIV prevention and treatment.

